# Therapeutic Potential of Phytocannabinoid Cannabigerol for Multiple Sclerosis: Modulation of Microglial Activation In Vitro and In Vivo

**DOI:** 10.3390/biom13020376

**Published:** 2023-02-16

**Authors:** Sigal Fleisher-Berkovich, Yvonne Ventura, Maya Amoyal, Arik Dahan, Valeria Feinshtein, Leenor Alfahel, Adrian Israelson, Nirit Bernstein, Jonathan Gorelick, Shimon Ben-Shabat

**Affiliations:** 1 Department of Clinical Biochemistry and Pharmacology, Ben-Gurion University of the Negev, Beer-Sheva 8410501, Israel; 2 Department of Physiology and Cell Biology, Ben-Gurion University of the Negev, Beer-Sheva 84710501, Israel; 3 ARO Volcani Center, Bet Dagan 50250, Israel; 4 Eastern Regional Research and Development Center, Judea Center, Kiryat Arba 90100, Israel

**Keywords:** cannabigerol, EAE, microglia, lipopolysaccharide neuroinflammation, nitric oxide

## Abstract

Multiple sclerosis (MS) is a widespread chronic neuroinflammatory and neurodegenerative disease. Microglia play a crucial role in the pathogenesis of MS via the release of cytokines and reactive oxygen species, e.g., nitric oxide. Research involving the role of phytocannabinoids in neuroinflammation is currently receiving much attention. Cannabigerol is a main phytocannabinoid, which has attracted significant pharmacological interest due to its non-psychotropic nature. In this research, we studied the effects of cannabigerol on microglial inflammation in vitro, followed by an in vivo study. Cannabigerol attenuated the microglial production of nitric oxide in BV2 microglia and primary glial cells; concomitant treatment of the cells with cannabigerol and telmisartan (a neuroprotective angiotensin receptor blocker) decreased nitric oxide production additively. Inducible nitric oxide synthase (iNOS) expression was also reduced by cannabigerol. Moreover, tumor necrosis factor-α (TNF-α), a major cytokine involved in MS, was significantly reduced by cannabigerol in both cell cultures. Next, we studied the effects of cannabigerol in vivo using a mice model of MS, experimental autoimmune encephalomyelitis (EAE). The clinical scores of EAE mice were attenuated upon cannabigerol treatment; additionally, lumbar sections of EAE mice showed enhanced neuronal loss (relative to control mice), which was restored by cannabigerol treatment. Altogether, the set of experiments presented in this work indicates that cannabigerol possesses an appealing therapeutic potential for the treatment of MS.

## 1. Introduction

Multiple sclerosis) MS) is the most prevalent chronic neuroinflammatory disease. It is an autoimmune and neurodegenerative disease defined by neuroinflammation, demyelination, axonal loss and neurodegeneration [1,2,3]. Microglia play a role in inflammatory responses, homeostasis, and tissue regeneration. Microglia have contributed to the development of autoimmune encephalomyelitis (EAE), in animal models of MS already in the initial stages [4]. Activated microglia present antigens and secrete major cytokines such as tumor necrosis factor-α (TNF-α). Additionally, they are involved in the demyelination and phagocytosis of the degraded myelin [5].

Studies showed that detected neurodegeneration is linked with acute demyelinating lesions [6,7,8], suggesting that inflammatory-related neuronal injury occurs early during the course of the disease. It has been recently shown that during experimental MS, a reversible form of focal axonal swelling, potentially evolving into axonal disruption, can be detected during the earlier phase of neuroinflammation, when the myelin sheet has not yet been affected [9]. The observed axonal damage was found to be more pronounced in brain areas with microglia and immune-cell infiltration. This may be explained by the release of reactive oxygen species such as nitric oxide by activated macrophages or microglia [9]. Interestingly, metabolomics studies revealed that biosynthesis of an inflammation inducer lipopolysaccharide (LPS) was found to be elevated in EAE mice, as confirmed by higher levels of LPS demonstrated in the brain. LPS results in the degeneration of myelin. It is noteworthy, again, that LPS is linked to neuroinflammation and microglial activation. 

The role of cytokines such as TNF-α in the underlying pathology of MS is supported by the observation that brains of MS patients have increased TNF-α levels at the site of active MS lesions [10]. TNF-α levels in the cerebrospinal fluid (CSF) of individuals with MS were elevated compared to healthy individuals, with TNF-α levels correlating with the severity of the lesions [11,12]. Nitric oxide (NO) and induced levels of inducible nitric oxide synthase (iNOS) enzyme, originating from resident CNS glial cells, are also observed during MS [13]. It is well-established that high amounts of NO in the brain can shift its role from being physiological to a neurotoxic factor [13,14,15].

Cannabinoids belong to a group of chemicals exemplified by Δ^9^-tetrahydrocannabinol (THC), the main psychoactive compound of *Cannabis sativa* [15]. The cannabinoid profile of *C. sativa* is under genetic and environmental control, with Δ^9^-THC, cannabidiol (CBD) and their precursor cannabigerolic acid (CBGA) being the most abundant ones [16]. 

The therapeutic effects of cannabinoids are antinociceptive, anti-epileptic, cardiovascular, anticancer, anti-inflammatory and more [17,18,19,20]. Recently, the endocannabinoid system was shown to be fully functional in the skin. As such, it has been studied for its ability to regulate skin cancer and inflammatory diseases such as psoriasis, acne, dermatitis, and scleroderma [17,18,19,20].

CBG, a main phytocannabinoid found in *C. sativa*, is attracting pharmacological interest because it is non-psychotropic and is abundant in some industrial hemp varieties. Research involving cannabinoids, such as THC and CBD, is currently receiving much attention. Combinations of CBD and THC slow disease progression in EAE mice. In human studies, the results are less encouraging and conflict with animal findings. Much less is known about CBG. The recent literature on CBG, a lipophilic resorcinol derivative, has revealed that its pharmacology addresses distinct therapeutic targets.

Previously, the beneficial effects of VCE-003, a quinone derivative of CBG, were shown in MS models [21,22]. The effect of CBG itself at 5 µM on microglial inflammation in vitro and its effect on EAE-induced neuroinflammatory response has been shown here for the first time, as far as we know. Moreover, CBG was shown to act more potently, here in vitro, than what was published previously [21]. 

Moreover, combination therapy has attracted a lot of attention in recent years, and new clinical studies have been centered on this topic. The goal of combination therapy is to minimize adverse effects and ideally to achieve a more efficient effect. This concept attempts to combine two therapeutic agents which act in different upstream pathways but affect similar downstream factors. Here, the combined role of CBG and telmisartan (an angiotensin 1 receptor blocker AT1R blocker (ARB)) or captopril (an angiotensin-converting enzyme inhibitor (ACEI)), both known to be neuroprotective, in regulation microglial NO release was measured. 

## 2. Materials and Methods

### 2.1. Cell Cultures

Murine BV2 microglia were kindly provided by Professor Rosario Donato (Department of Experimental Medicine University of Perugia, Italy) [23]. Microglia were maintained in RPMI-1640 medium with 10% fetal calf serum (FCS), streptomycin (100 µg/mL), penicillin (100 U/mL) and L-glutamine (4 mM) in 5% CO_2_ humidified air incubator at 37 °C. Culture medium was replaced twice a week. 

For each in-vitro experiment, serum-free medium (SFM) was added to the cells 4 h before the experiment initiation. Thereafter, microglia were treated with SFM containing 0.1% bovine serum albumin (BSA) and HEPES buffer (10 mM at pH 7.4) in the absence or presence of test agents for 22 h. All culture media were purchased from Biological Industries (Kibbutz Beit-Haemek, Israel). CBG, LPS from *Escherichia coli* O55:B5 and poly-L-lysine were purchased from Sigma Aldrich (Rehovot, Israel). 

Primary neonatal rat glial cell cultures were prepared from whole brains of 1-day-old Wistar rats, and grown according to accepted protocols [23,24]. Immunocytochemistry studies as previously described [24,25] revealed that these cultures contain about 80% astrocytes and about 20% microglia. 

### 2.2. Cell Viability

Cells were seeded at a density of 1 × 10^4^ cells per well in 96-well plates and cultivated overnight in complete RPMI-1640 medium. Thereafter, cells were treated with the respective test agents, as described above. Subsequently, XTT reagent was mixed with the activation reagent, at a ratio of 50:1 according the manufacture’s protocol (Biological Industries, Kibbutz Beit-Haemek, Israel) and was added to each well in a 1:2 ratio. Absorbance was measured at 450 nm against a reference wavelength at 650 nm after 1 h incubation at 37 °C using a microplate reader (Model 680, Bio-Rad, Hercules, CA, USA). 

### 2.3. Determination of NO Levels (Griess Reaction)

Nitrite levels in culture media, as an indicator for NO release, were determined by an established assay using Griess reagent (Sigma Aldrich, Rehovot, Israel). 

### 2.4. Determination of TNF-α Levels (ELISA)

TNF-α levels in the culture media were determined using enzyme-linked immunosorbent assay (ELISA) kit (BD Biosciences, San Diego, CA, USA) according to the manufacturer’s protocol.

### 2.5. Western Blot Analysis

Forty (40) μg of protein from whole cell lysate were loaded on 7.5% polyacrylamide-SDS gels and blotted on a nitrocellulose membrane. After blocking with 4% BSA for 90 min, membranes were incubated overnight at 4 °C with rabbit anti-iNOS antibody (130 kDa) (1:500, Cayman Chemicals, Ann Arbor, MI, USA). Upon washing, the blots were incubated for 90 min in the corresponding conjugated donkey anti-rabbit antibody (1:10,000, GE Healthcare, Buckinghamshire, UK). The position of the individual protein was detected after exposure to the ChemiDocTM XRS+ (Bio-Rad Laboratories, Hercules, CA, USA) image system. Band-intensity analysis was performed using a computerized image analysis system (ImageJ software, version 1.40C, NIH). Protein quantity was normalized to β-actin protein (40 kDa) level measurements using mouse monoclonal anti-β-Actin−Peroxidase antibody (1:20,000, Sigma Aldrich, Israel). 

### 2.6. Active MOG-Induced EAE Model

Female eight-week-old C57BL/6 mice (Envigo, Jerusalem, Israel) were immunized with myelin oligodendrocyte glycoprotein (MOG) [peptide 35–55] (AnaSpec, Fremont, CA, USA). Each mouse was injected subcutaneously (s.c.) into 2 sites on the back, adjacent to each of the hind limbs (total volume 200 μL), with 200 μg MOG emulsified with a mixture of 200 μg/mL killed *Mycobacterium tuberculosis* H37RA (Difco, Detroit, MI, USA) in complete Freund’s adjuvant (BD Biosciences, San Jose, CA, USA). Thereafter, each animal was injected intraperitoneally (i.p.) with 400 ng/mL reconstituted pertussis toxin (ENCO, Petah Tikva, Israel), which was repeated two days after the initial immunization. National and institutional guidelines for the care and use of laboratory animals were followed.

After immunization, the mice were evaluated for neurological scores as follows: 0 normal; 0.5 mild ataxia of the hind limb; 1 decreased tail tone; 1.5 righting reflex within 3 s; 2 righting reflex between 4 and 7 s; 2.5 righting reflex between 7 and 10 s; 3 hind limbs paralysis or absolute loss of righting reflex; 4 front and hind limbs paralysis (n = 12, 6 of them obtained only MOG and 6 of them obtained MOG+CBG). From day 12 post-immunization treatment, administrations were performed i.p. for four consecutive days. Control EAE mice received only the vehicle solution composed of Tween-20:ethanol:saline at a ratio of 1:1:8 (n = 4). CBG EAE mice received CBG (10 mg/kg) dissolved in the vehicle solution (n = 6). The experiment was terminated by euthanizing the mice, followed by cardiac perfusion. Thereafter, spinal columns were fixed in 4% formaldehyde at 4 °C overnight and cryoprotected in 20% sucrose for 48 h at 4 °C. Then, spinal cords were dissected and mounted in OCT (Scigen Scientific, Gardena, CA, USA), snap frozen at −40 °C and, finally, stored at −80 °C. 

### 2.7. Immunohistochemistry

Free-floating sections (30 µm thick) were blocked for 1 h in blocking buffer (Antibody diluent (GBI Labs, Bothell, WA, USA) with 0.5% Triton), and immunostained overnight at 4 °C with antibodies diluted in antibody diluent with 0.15% Triton. The following antibodies were used: mouse anti-neuronal nuclei antigen (NeuN, 1:100) (Millipore, Temecula, CA, USA), monoclonal anti-GFAP (1:400) (Millipore, Temecula, CA, USA), rat anti-CD4 (1:50) (BD Pharmingen, San Diego, CA, USA) and rabbit anti-Iba1 (1:1000) (Fujifilm Wako, Richmond, VA, USA). The next morning, sections were washed three times with 0.05 % Tween 20 in PBS, then incubated for 1 h at room temperature with a fluorescent-conjugated secondary anti-rat, anti-mouse or anti-rabbit antibody (1:200, Alexa Fluor 647; Jackson). Sections were mounted on slides with Immu-Mount (Thermo Scientific, Ann Arbor, MI, USA). Images were obtained using the Olympus FluoView FV1000 confocal microscope (Olympus, Hamburg, Germany). 

### 2.8. Statistical Analysis

Experimental data are presented as the mean ± standard error of mean (SEM). For significance assessment between groups, one-way analysis of variance (ANOVA) and post-hoc multiple comparison test (Tukey–Kramer multiple comparison test) were performed. Statistical significance was considered at *p* < 0.05.

## 3. Results

### 3.1. Cell Viability

**Figure 1:**
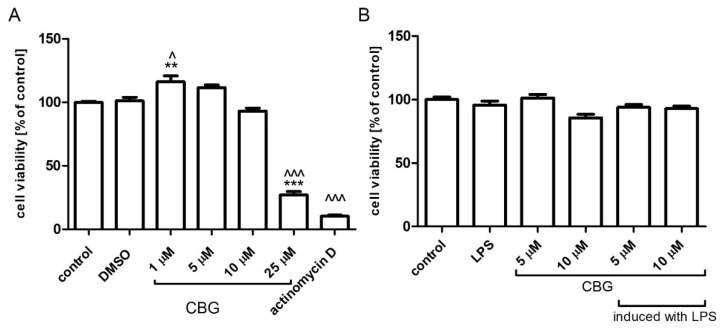
Cell viability following treatment with increasing CBG concentrations. BV2 microglia (**A**) and rat primary mixed glial cells (**B**) were pre-incubated with SFM for 4 h. Then, CBG was added for 22 h. Cell viability was determined by XTT proliferation assay. Data are presented as means ± SEM and are representatives of two independent experiments (n = 6). Statistical significance was determined using one-way ANOVA, followed by a Tukey–Kramer multiple comparison test. ** *p* < 0.01 vs. control; *** *p* < 0.001 vs. control; ^ *p* < 0.05 vs. DMSO; ^^^ *p* < 0.001 vs. DMSO.

We examined the viability of BV2 microglial cells treated with concentrations of 1, 5, 10 and 25 μM CBG. While 1 μM CBG significantly increased cell viability as compared to control or DMSO-treated cells, CBG at 5, 10 μM did not change the viability of BV2 cells. The highest concentration of CBG used, namely, 25 μM, attenuated cell viability by 70%. Almost a complete reduction in cell viability was observed using the transcription inhibitor actinomycin D (Figure 1A). No significant differences in cell viability were found between control and LPS-treated (0.5 µg/mL) with or without CBG treatment in primary rat glial cells (Figure 1B).

### 3.2. NO Release and TNF-α Production

**Figure 2:**
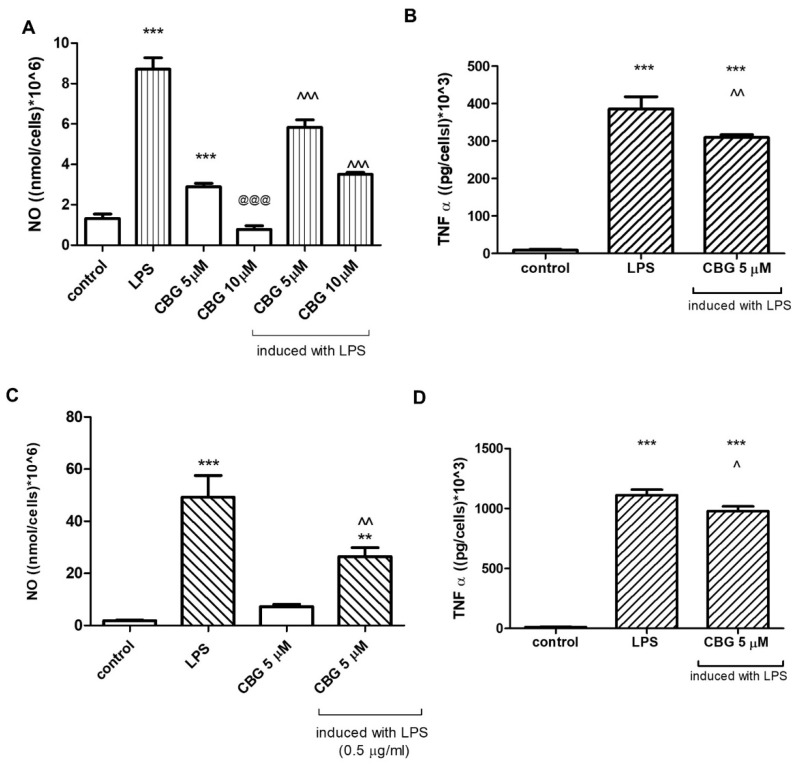
CBG decreased NO (**A**,**C**) and TNF-α (**B**,**D**) production by LPS-induced BV2 microglia (**A**,**B**) and in primary rat glial cells (**C**,**D**). Cells were pre-incubated with SFM for 4 h and then incubated for 22 h with 7 ng/mL LPS (**A**,**C**) and 0.5 μg/mL LPS (**B**,**D**) in the presence or absence of 5 or 10 μM CBG. Culture media were analyzed for NO (**A**,**C**) and TNF-α (**B**,**D**) levels and normalized to cell counts. Means ± SEM of representatives of three (**A**,**C**) or two (**B**,**D**) independent experiments are presented (n = 6). One-way ANOVA and a Tukey–Kramer multiple comparison test were performed for statistical significance. *** *p* < 0.001 vs. control; ** *p*<0.005 vs. control; @@@ *p* < 0.001 vs. CBG 5 μM; ^^^ *p* < 0.001 vs. LPS; ^^ *p* < 0.005 vs. LPS; ^ *p* < 0.05 vs. LPS.

**Figure 3:**
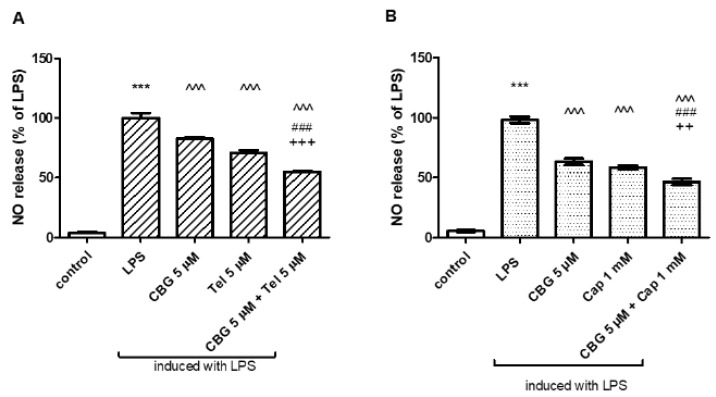
CBG in combination with telmisartan (**A**) but not captopril (**B**) additively decreased NO production in LPS-induced BV2 microglia. Cells were pre-incubated with SFM for 4 h and then incubated for 22 h with 7 ng/mL LPS in the presence of the specific compounds. Culture media were analyzed for NO levels and normalized to cell counts. Means ± SEM of representatives of two–three independent experiments are presented (n = 6). One-way ANOVA and a Tukey–Kramer multiple comparison test were performed for statistical significance. *** *p* < 0.001 vs. control; ^^^ *p* < 0.001 vs. LPS; ### *p* < 0.001 vs. CBG; +++ *p* < 0.001 vs. Tel; ++ *p* < 0.005 vs. Cap.

Treatment with LPS (7 ng/mL) stimulated NO release from BV2 cells into the culture media, while CBG dose dependently (5, 10 μM) reduced LPS-induced NO production (Figure 2A). Without LPS induction, the basal NO level was significantly induced by 5 μM CBG. NO levels were significantly reduced when CBG concentration was increased (10 μM) (Figure 2A). LPS-induced TNF-α synthesis was significantly decreased by 5 μM CBG (Figure 2B). In addition to BV2 microglia, also in rat primary mixed glial cells, LPS (0.5 µg/mL) significantly induced NO and TNF-α production. Treatment with 5 μM CBG reduced significantly the NO release (Figure 2C), while TNF-α production was attenuated by 13% after the application of 5 μM CBG (Figure 2D). Moreover, we examined the synthesis of NO in BV2 cells stimulated by LPS (7 ng/mL) and treated with the neuroprotective agents telmisartan (5 μM) (Figure 3A) or captopril (1 mM) (Figure 3B) with or without CBG (5 μM) (A). While LPS significantly enhanced NO synthesis as compared to non-stimulated cells (control), telmisartan (5 μM) and CBG (5 μM) attenuated this effect by 17% and 29%, respectively. Concomitant CBG and telmisartan treatment (Figure 3A) additively decreased NO production in LPS-induced BV2 microglia. Concomitant CBG and captopril treatment decreased NO production as well; this effect was higher than the effect of each agent separately, but was lower than the additive degree (Figure 3B).

### 3.3. iNOS Protein Levels

**Figure 4:**
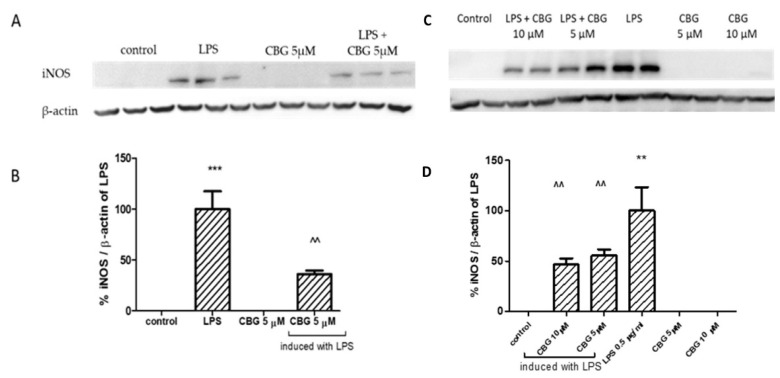
CBG decreased iNOS expression in LPS-induced BV2 microglia (**A,B**) and primary rat glial cells (**C,D**). Immunodection of iNOS with or without induction of LPS (7 ng/mL) in the presence or absence of CBG (5 μM) for BV2 (**A,B**) and with or without induction of LPS (0.5 µg/mL) in the presence or absence of CBG (5 and 10 μM) for primary rat glial cells (**C,D**). Forty (40) μg of protein from whole cell lysate was loaded on 7.5% polyacrylamide-SDS gels and blotted on a nitrocellulose membrane. Protein quantities were normalized to β-actin levels. Analysis of iNOS was performed using antibodies against iNOS (130 kDa) and β-actin (40 kDa). Band intensity analysis was performed using ImageJ software. Results are representative of two independent experiments and are presented as means ± SEM (n = 4–6). *** *p* <0.001 vs. control; ** *p* <0.005 vs. control; ^^ *p* < 0.005 vs. LPS.

The 22 h exposure to LPS in BV2 cells (7 ng/mL) or primary glial cells (0.5 μg/mL) resulted in a robust increase in iNOS protein levels (Figure 4), by more than 90% as compared with the control. Yet, 22 h incubation with LPS together with 5 μM CBG significantly reduced iNOS expression levels by 65% in BV2 cells (Figure 4A,B) and by 45% in primary rat glial cells (Figure 4C,D), respectively. Higher application of 10 μM CBG in the primary rat glial culture reduced iNOS level by 50%. CBG alone did not alter iNOS protein expression level. 

### 3.4. EAE Studies

**Figure 5:**
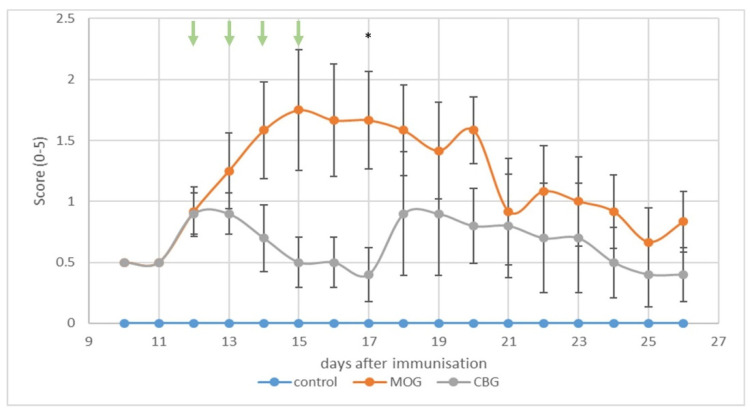
Evaluation of the clinical score over time of C57BL/6 female mice immunized with MOG. Mice were treated once a day with CBG (10 mg/kg) or vehicle (Tween 20:ethanol:saline; 1:1:8) for four consecutive days starting on day 12 p.i. (indicated by green arrows). The results are shown as mean  ±  SEM of the experimental groups (1 experiment n = 4–6 mice in each group). One-way ANOVA followed by Tukey–Kramer multiple comparison post-test was performed. * *p* < 0.05 vs. vehicle.

In vivo, we investigated the potential of i.p. CBG at a clinically relevant dose on neurological scores of EAE mice (Figure 5). Immunization with MOG induced EAE in mice, giving a mean onset on day 9 post-immunization (p.i.). All MOG-treated mice developed a disease with a mean score of 1.75. By contrast, the clinical manifestations of EAE were attenuated in mice receiving four injections of CBG (10 mg/kg) at days 12 to 15 upon immunization) Figure 5). In the mice that received CBG, a first peak in disease severity appeared between days 12 and 13 with a subsequent decline until day 17 post-immunization. Thereafter, a second peak followed at day 18 which slowly declined until the end of the experiment. The mean severity score in the group receiving CBG was always less than 1 throughout the course of the experiment from day 9 to day 26. 

### 3.5. Immunohistochemistry Evaluations

**Figure 6:**
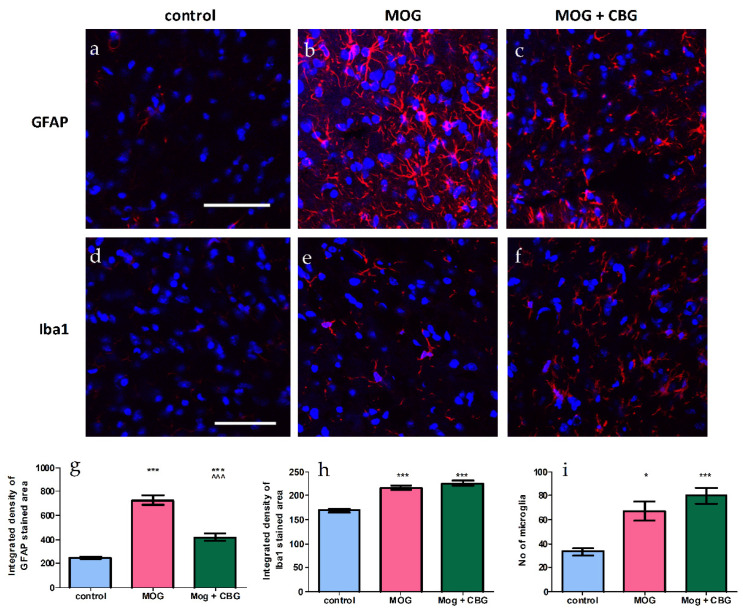
Immunohistochemistry of spinal cords of non-treated and EAE mice. Lumbar sections were stained with GFAP (**a**–**c**) and Iba1 (**d**–**f**) (red), and counterstained for nuclei with DAPI (blue). Representative lumbar layers from the mice groups are presented (one experiment, n = 4–6 in each group). Immunodetections were quantified and plotted as integrated density for each antibody (**g**,**h**). The average number of microglia are presented in (**i**). One way ANOVA followed by a Tukey–Kramer multiple comparison test were performed to determine statistical significance. * *p* < 0.05 vs. control, *** *p* <0.001 vs. control; ^^^ *p* <0.001 vs. MOG. The scale bar is 50 µm.

**Figure 7:**
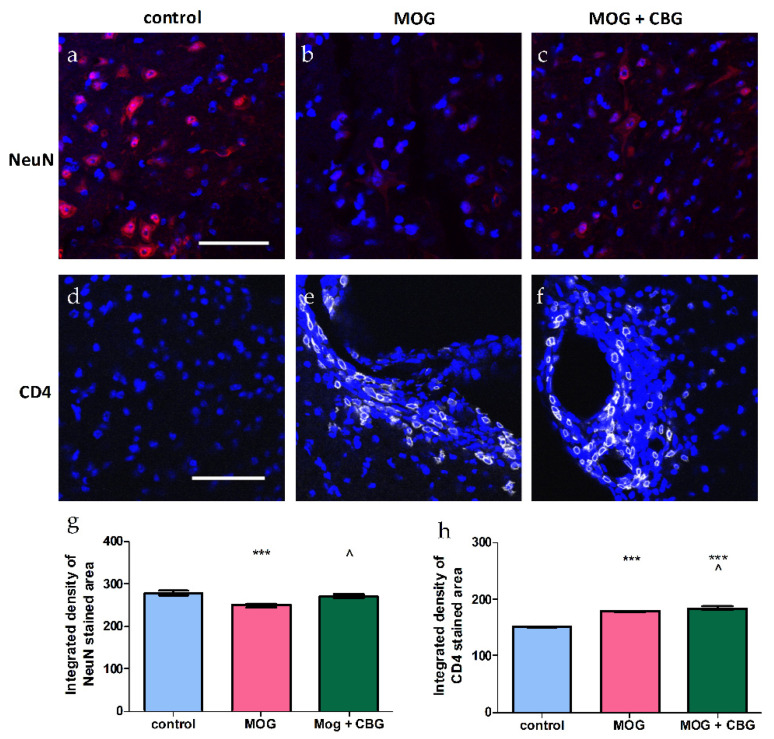
Immunohistochemistry of spinal cords of non-treated and EAE mice. Lumbar sections were stained with NeuN (**a**–**c**) (red) and CD4 (**d**–**f**) (white) and counterstained for nuclei with DAPI (blue). Representative lumbar layers from the mice groups are presented (n = 4–6 for each group). Immunodetections were quantified and plotted as integrated density for each antibody (**g**,**h**). One way ANOVA followed by a Tukey–Kramer multiple comparison test were performed to determine statistical significance. *** *p* < 0.001 vs. control; ^ *p* < 0.05 vs. MOG. The scale bar is 50 µm.

The effects of i.p. administration of CBG on astrocytosis, demonstrated by GFAP staining (Figure 6a–c,g); microgliosis, demonstrated by Iba1 staining (Figure 6d–f,h,i); and neuronal loss, demonstrated by NeuN staining (Figure 7a–c,g) and CD4 staining (Figure 7d–f,h) were investigated in non-treated and MOG-treated (EAE) mice by immunohistochemistry in lumbar sections of spinal cords. Lumbar sections of control mice showed low GFAP, Iba1 and CD4 staining. By contrast, lumbar sections of EAE mice exhibited induced levels of astrogliosis, microglial activation and CD4 expression when compared to control mice. As expected, neuronal loss was enhanced in EAE mice as compared with control mice, but was restored by 10 mg/kg CBG when administrated for four consecutive days. The same CBG treatment significantly reduced the areas stained for GFAP in EAE mice, while CD4 and Iba1 expression was not changed upon CBG treatment. 

## 4. Discussion

MS is accompanied by activation of glia [26]. In fact, microglia play a dual role, sometimes inducing inflammation, but in other cases inducing repair by clearing myelin and cell debris [27,28]. Astrocytes are also a major component of MS plaques [29] well positioned to enhance inflammation by cytokines such as TNF-α and free radicals such as NO, but they may also limit damage by providing metabolic support to axons [30].

Glial NO can rapidly react with a superoxide anion to form peroxynitrite (ONOO^-^), one of the most deleterious reactive oxygen species [31]. Peroxynitrite plays an important role in the pathology of demyelinating diseases, such as MS [32,33,34].

In the present study, we demonstrate the anti-inflammatory and anti-oxidative effects of CBG, by itself, as revealed by the attenuation of BV2 microglial production of NO, iNOS and TNF-α stimulated by LPS (7 ng/mL) in BV2 cells and in primary glial cultures induced by LPS (0.5 µg/mL). CBG in both models of inflammation demonstrated higher (two–five-fold) potency in attenuating microglial inflammatory response as compared to data published previously [21]. In recent years, a series of CBG quinone derivatives such as VCE-003, which act as PPAR-gamma activators, showing low affinity for cannabinoid receptors, have been characterized [21,35]. As shown by Gugliandolo et al., VCE-003 reduced the expression of the iNOS protein in LPS and IFN-γ-treated BV2 microglia [21]. In addition, VCE-003.2 reduced iNOS mRNA in BV2 microglia exposed to high levels of LPS [22]. Interestingly, CBG such as CBD may also be converted to CBG-hydroxy-quinone (a precursor of VCE-003.2) during liver metabolism, explaining at least part of the neuroprotective effect of CBG in vivo.

Female mice were chosen to be studied in this EAE model, since autoimmune diseases are more prevalent in females than males. This discrepancy was also found in animal models. Increased spinal-cord lesions and demyelination are shown in female EAE mice vs. males. 

This study provides evidence that CBG by itself attenuated the neurological deficit score in EAE mice. It also reduced astrogliosis and decreased neuronal loss in EAE mice. An increase in CD4 T-cell populations was also observed upon the CBG treatment of EAE mice. The major pro-inflammatory CD4 T-cells associated with autoimmune diseases, including MS, are the Th1 CD4 T-cells. These cells secrete IFN-γ and TNF-α [36,37]. Autoimmune diseases are also associated with Th2 CD4 T-cells, which are induced by IL-4 [38]. Th2 CD4-related cytokines, such as IL-4 and IL-10, are anti-inflammatory and improve symptoms in MS patients. Th1 CD4 cytokines have been shown to increase inflammation, and lead to disease progression and the worsening of symptoms [39,40]. Th2 and Th1 cytokines can cross-inhibit each other and the progression of disease may depend on the balance between both types of cytokines [39,40]. In this study, we saw an improvement in EAE symptoms, suggesting the occurrence of a shift toward a Th2 cytokine anti-inflammatory response. The protective effects observed here may also be due to changes in microglial inflammatory functions. An analysis of the CD4+ T cell phenotypes in EAE spinal cords would help determine the anti-inflammatory effects of CBG. Our data correlates with other studies using different in-vivo models. Valdeolivas has shown the anti-inflammatory role of CBG in two experimental models of Huntington’s disease [41]. Beneficial effects of CBG were also shown in experimental inflammatory bowel disease [42]. VCE-003 alleviated neuroinflammation and motor deficits in the viral TMEV model of MS. VCE-003 also suppressed immune responses and neuroinflammation in EAE mice [35]. In summary, based on its antioxidant and anti-inflammatory activities, CBG may hold great promise as an antioxidant agent and, therefore, may be used in clinical practice as a new approach in oxidative-stress-related disorders. An additional intriguing point was the potential therapeutic interest in the combined administration of telmisartan/captopril and CBG. ARBs such as telmisartan and ACEIs such as captopril were shown to be neuroprotective by us and others. Both telmisartan and captopril in combination with CBG significantly reduced NO synthesis compared to each compound by itself. Telmisartan, but not captopril, acted additively with CBG to inhibit NO production in LPS-stimulated microglia. This suggests that distinct cellular signaling pathways in microglia may be activated by telmisartan and captopril. Further work is warranted to determine the mechanism of action of CBG and the compounds tested here, at both receptor and intracellular signaling levels, to enhance our ability to develop novel safe and effective treatment strategies for microglial inflammation and neurodegeneration. 

## Data Availability

The data presented in this study are available on request from the corresponding author. The data are not publicly available due to privacy issues.

## Abbreviations

Lipopolysaccaride (LPS), Cannabigerol (CBG), Nitric Oxide (NO), Serum free medium (SFM), Experimental autoimmune encephalomyelitis (EAE), multiple sclerosis) MS), inducible nitric oxide synthase (iNOS), cerebrospinal fluid (CSF).
